# An Insight into Potential Pharmacotherapeutic Agents for Painful Diabetic Neuropathy

**DOI:** 10.1155/2022/9989272

**Published:** 2022-01-27

**Authors:** Zunaira Qureshi, Murtaza Najabat Ali, Minahil Khalid

**Affiliations:** Department of Biomedical Engineering and Sciences, School of Mechanical and Manufacturing Engineering, National University of Sciences and Technology, H-12, 44000 Islamabad, Pakistan

## Abstract

Diabetes is the 4^th^ most common disease affecting the world's population. It is accompanied by many complications that deteriorate the quality of life. Painful diabetic neuropathy (PDN) is one of the debilitating consequences of diabetes that effects one-third of diabetic patients. Unfortunately, there is no internationally recommended drug that directly hinders the pathological mechanisms that result in painful diabetic neuropathy. Clinical studies have shown that anticonvulsant and antidepressant therapies have proven fruitful in management of pain associated with PDN. Currently, the FDA approved medications for painful diabetic neuropathies include duloxetine, pregabalin, tapentadol extended release, and capsaicin (for foot PDN only). The FDA has also approved the use of spinal cord stimulation system for the treatment of diabetic neuropathy pain. The drugs recommended by other regulatory bodies include gabapentin, amitriptyline, dextromethorphan, tramadol, venlafaxine, sodium valproate, and 5 % lidocaine patch. These drugs are only partially effective and have adverse effects associated with their use. Treating painful symptoms in diabetic patient can be frustrating not only for the patients but also for health care workers, so additional clinical trials for novel and conventional treatments are required to devise more effective treatment for PDN with minimal side effects. This review gives an insight on the pathways involved in the pathogenesis of PDN and the potential pharmacotherapeutic agents. This will be followed by an overview on the FDA-approved drugs for PDN and commercially available topical analgesic and their effects on painful diabetic neuropathies.

## 1. Introduction

A number of reviews on peripheral neuropathy have been published in general and on diabetic neuropathic pain, in particular [1]. Most of these reviews gave us an insight about the classification and mechanism of painful diabetic neuropathy (PDN) which can help us in developing correct diagnosis and successful treatment against it. An ideal treatment for PDN can be defined as those compounds that prevent the progressive loss of nerve function and improve the symptoms with minimal side effects. Several combinations of drugs have been approved by the Food and Drug Authority (FDA), American Academy of Neurology (AAN), American Association of Clinical Endocrinologists (AACE), American Diabetes Association (ADA), European Federation of Neurological Sciences (EFNS), and National Institute of Clinical Examination (NICE) which have been proven partially fruitful in managing symptoms of PDN. Many other drugs are under trials, and some drugs have been withdrawn from the market as they pose serious health risks in long-term usage [2]. Management of PDN is still challenging because of its complex and underassessed pathophysiology.

Neuropathic pain is defined by International Association for the Study of Pain (IASP) as “pain caused by a lesion or disease of the somatosensory nervous system” [3]. Diabetes mellitus (DM) is a complex metabolic disorder which is characterized by high blood glucose levels, due to inadequate insulin secretion by the pancreas or inability of target cells to reuptake glucose from the blood [4]. Diabetic peripheral neuropathy (DPN) is one of the consequences of diabetes which is accompanied by other risks of getting cardiovascular, peripheral, and cerebrovascular diseases [2]. Globally, diabetes was the 7th leading cause of death in 2016 [5], and according to “The Lancet Commission on Diabetes: using data to transform Diabetes Care and Patient Lives” report published by International Diabetes Care, the number of people affected by DM is 463 million which is 5.9% of total world population, and it is expected that by 2045, 629 million (6.3%) people are to be affected by DM [6]. In general, peripheral diabetic neuropathy and more specifically painful peripheral diabetic neuropathy are the main causes that deter the quality of life and lead to high morbidity rate. A study conducted by Baxi et al. on larger population shows that 28.85% of the diabetic population suffered from DPN and out of which 88% of the population showed pain symptoms [7].

In this paper, the metabolic pathways involved in the pathogenesis of PDN and the potential targets for its treatment will be discussed. Followed by which, a brief overview of the topical agents available in the market and the FDA-approved drugs will be provided. In the end, the nonpharmacological treatment modalities including the recently FDA-approved spinal cord stimulation system will also be discussed.

## 2. Methodology

A comprehensive literature review was undertaken, incorporating articles from electronic databases (Google Scholar and PubMed) by using keywords like “diabetes, painful diabetic neuropathies, pathological mechanisms, anticonvulsants drugs, FDA/EU approved drugs etc.” and other relevant lists of articles with author name. Explanatory data from those articles was taken and incorporated in this review in a descriptive manner. All the preclinical and clinical studies mentioned in this review solely focus on painful diabetic neuropathy and not on any other form of neuropathies.

## 3. Classification

**Figure 1:**
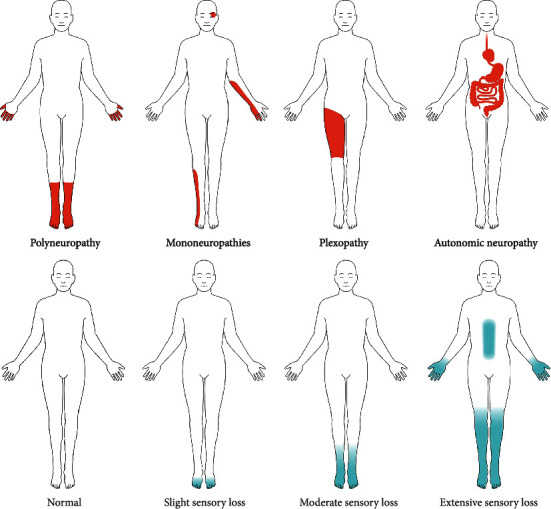
Clinical appearance of the most prevalent type of diabetic neuropathy and progression of sensory loss over the course of DSPN. Adapted from [13] (CC BY 4.0)

Diabetic neuropathy can be classified into two broad categories: diffuse and focal neuropathies. Diffuse neuropathies branch into diabetic peripheral neuropathy (DPN) and diabetic autonomic neuropathy (DAN). Peripheral neuropathies usually affect the nerves present in the extremities. Both small and large nerve fibers are affected by DPN. Damage to large nerve fibers interferes with the body movement and body position whereas demyelination of smaller nerve fibers in the peripheral region causes dysesthesias and paresthesia linked with neuropathic pain [8]. There are numerous types of peripheral diabetic neuropathy, the most common being the distal symmetry diabetic sensorimotor polyneuropathy (DSPN). DSPN accounts for 75-90% of all the diabetic neuropathy cases and can present itself as either painful (pDSPN) or nonpainful. The clinical appearance of the most prevalent forms of diabetic neuropathy, as well as the progressive sensory loss that occurs throughout the course of DSPN, are depicted in Figure 1. In pDSPN, burning, stabbing, numbing, or deep ache pains are felt in the periphery where multiple neurons are affected [9]. Oxidative stress caused by diabetes due to underlying pathogenic events results in the sensory sensitization and demyelination of neurons in the periphery. A study conducted on rats showed that metabolic flux is the primary cause of demyelination and progression of peripheral neuropathy [10]. Diffused autonomic neuropathy is associated with poor lifestyle and progresses slowly. It affects the physiological systems which are controlled by autonomic nervous system, i.e., cardiovascular, gastrointestinal, and genitourinary [1].

Mononeuropathies affect the medial, ulnar, and lateral popliteal nerves [11]. Radiculoplexopathy affects the motor neurons in the lumbosacral region [12]. Sensory losses are difficult to detect and may remain hidden for a longer period. Both the above-mentioned neuropathies come under focal and multifocal neuropathies which are less common as compared to peripheral neuropathies.

Painful distal symmetrical diabetic sensorimotor polyneuropathy (pDSPN) is mostly referred to when painful diabetic neuropathy (PDN) is stated, as it is the most common subtype of PDN. The other subtypes of PDN include mononeuritis multiplex, mononeuropathy, small fiber neuropathy, diabetic lumbosacral radiculoplexus neuropathy, and treatment-induced neuropathy. pDSPN or PDN is defined as “pain caused by a lesion of the somatosensory system attributed to diabetes” [13]. This article will focus on painful diabetic neuropathy, and hereinafter, PDN will indicate pDSPN throughout this text.

## 4. Systemic/Localized/Topical Treatments

Systemic therapies affect the entire body whereas localized therapies target the specific injured areas. Multiple drugs are used in systemic treatment and management, but there are a few drugs for the local management of PDN. The anticonvulsants, antidepressants, and opioids recommended for PDN are given as systemic therapies [14, 15]. These drugs are administered orally which provides temporary relief from pain but exhibits adverse side effects, e.g., dizziness, dry mouth, and muscle weakness [16]. Topical treatment options are available in the form of 5% lidocaine patch, capsaicin, and nitric oxide spray to manage pain symptoms. Recently, topical treatments have been in the spotlight as they pose lesser health risk compared to orally administered treatment options, but they are still not efficacious enough [17].

## 5. Metabolic Pathways of Painful Diabetic Neuropathy (PDN) and Potential Therapeutic Agents

**Figure 2:**
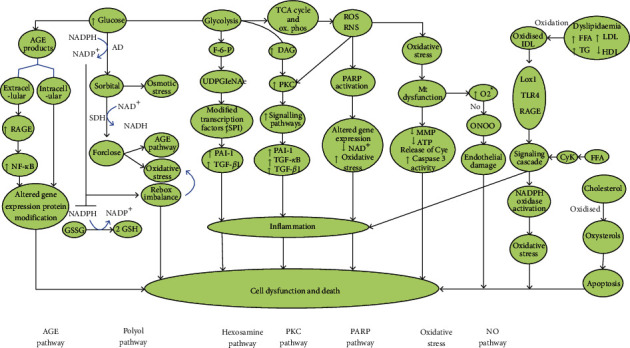
Important pathophysiological pathways involved in diabetic neuropathy [47]

The potential therapeutic agents for PDN include the inhibitors of signaling molecules or activators of suppressor signals indicated in the pathophysiology of painful diabetic neuropathy (Figure 2).

### 5.1. Polyol Pathway

Polyol pathway comprises two main enzymes named as aldose reductase (AR) and sorbitol dehydrogenase which are responsible for the metabolism of excessive glucose [18]. AR converts glucose in the presence of NADPH cofactor into sorbitol, which is in turn converted into fructose in the presence of NAD^+^. A small amount of glucose concentration gets metabolized through this pathway as AR exhibits lower affinity for glucose. In hyperglycemic state, the excess glucose is consumed using this pathway resulting in an increase in NADPH level and reductive stress [19]. This stress along with the mitochondrial dysfunction impairs the Schwann cell function causing compromised myelination, abnormal neurotrophic support to the axon, and therefore a loss of axon function [20]. Because of increased sorbitol and fructose concentration, events like reduced efflux of myoinositol, inhibition of ATP synthesis, and a resultant compromised Na^+^ and K^+^ ATPase activity are observed. Additionally, axon-glia dysfunction and reduced nerve conduction velocity because of structural degeneration of nerves are also examined. It also causes the downregulation of glutathione reduction pathway which causes the accumulation of free radical and peroxides, thus aggravating the nerve damage and resulting in NO-mediated vasodilation [21].

Inhibition of Aldose reductase pathway is one of the primary targets for many therapeutic agents. Epalrestat is reversible aldose reductase inhibitor which has been proven effective against peripheral diabetic neuropathy. Clinical studies demonstrated the alleviation of spontaneous pain in the lower limbs of 48.6% diabetic patients [22]. Epalrestat, a carboxylic derivative, has been found beneficial in inhibiting polyol pathway and shielding against nerve damage with no side effects until recently a study conducted by Le et al. highlighted the induction of liver fibrogenesis due to increased oxidative stress [23, 24]. Studies conducted on tolrestat, zenarestat, and sorbinil suggested their withdrawal from human use because of their adverse effects, i.e., liver dysfunction, increased creatinine levels, and serious hypersensitivity, respectively [25]. Other aldose reductase inhibitors (ARIs) (fidarestat, ponalrestat, zopolrestat, and lidorestat) have been used for the management of diabetic complication, but due to their adverse effects, they are not capable of producing desired outcomes (Singh [26]). Ranirestat is one of the ARI that has advanced to human trials, because it demonstrated positive results in improving the nerve conduction velocity, sensory perception, and nerve fiber density in patient suffering from diabetic polyneuropathy, but the effects on PDN are yet to be investigated [27, 28].

### 5.2. Hexosamine Pathway

One of the glycolysis product fructose-6-phosphate is converted into glucosamine-6-phophate in the presence of glutamine fructose-6-phosphate amidotransferase (GFAT) which is in turn converted into uridine-5-diphosphate-N-acetyl glucosamine [29]. In type 2 diabetes, glutamine fructose-6-phosphate amidotransferase (GFAT) is involved in insulin resistance and hyperinsulinemia, while the end product of this pathway, uridine-5-diphosphate-N-acetyl glucosamine, causes gene transcription factor specificity protein 1 (Sp1) to increase which then activates the transforming growth factor beta (TGF-*β*) and plasminogen activator inhibitor-1 (PAI-1), responsible for damaging endothelial cells and prompting smooth muscle cell division. This further leads to microvascular complications, especially diabetic neuropathy which damages the vessels supplying blood to the nerves [19, 30].

Benfotiamine reduces the production of advanced glycosylation end products by impeding glucose metabolism via the hexosamine pathway. In 2008, a study was conducted to evaluate the safety and efficacy of benfotiamine in diabetic polyneuropathy patients. It was observed that the drug ameliorated pain to a greater extent, but the results were not statistically significant [31]. Recently, the effect of coadministration of benfotiamine and alpha lipoic acid (free radical scavenging drug) was assessed in a total of 120 patients diagnosed with PDN. The drugs were orally administered with the coadministration test group given a dose of 300 mg/day and 600 mg/day respectively. It was found that the coadministration of these drugs exhibited a more pronounced efficacy than the monotherapy of the individual drugs [32].

### 5.3. Protein Kinase C Pathway

Multiple studies have confirmed the involvement of protein kinase C (PKC) in diabetic neuropathy [33]. Protein kinase C consists of serine/threonine protein kinase family which are responsible for many cellular processes and affect signaling transduction cascade associated with apoptosis, differentiation, and proliferation. In general, PKC isoforms in humans are divided into three subtypes; classical, atypical, and novel, depending on the secondary messenger they require for activation. Upregulation of diacylglycerol and Ca^+2^ activate conventional PKC isoforms (*α*, *β*I, *β*II, and *γ*); on the other hand, only diacylglycerol (DAG) is responsible for the activity of novel isoforms of PKC (*δ*, *ε*, *η*, and *θ*). Atypical isoforms get activated in the absence of diacyl glycerol (DAG) and Ca^+2^ and consist of protein kinase M*ζ* and *ι*/ *λ* isoform. According to Borghini et al., protein kinase isoforms *α*, *β*I, *β*II, *γ*, *ε*, and *δ* are present in the nerves [34].

In the diabetic condition, PKC pathway originates from the glyceraldehyde-3-phosphate of the glycolysis pathway. As part of PKC pathway, the glyceraldehyde-3-phosphate is converted to dihydroxyacetone which is then converted into glycerol-3-phosphate and ultimately into DAG. DAG and/or advanced glycation end products (AGEs) activates the PKC which then upregulates a number of signaling cascades by protein phosphorylation. PKC is involved in the activation of vascular endothelial growth factor (VEGF), plasminogen activator ihibittor-1 (PAI-1), transforming growth factor beta-1 (TGF-*β*), and nuclear factor kappa-B (NF-*κ*B) which leads to microvascular complications. PKC also down regulates Na^+^/K^+^ ATPase causing normalization of sciatic nerve conduction velocity and nerve blood flow (Simran et al. 2019). It has been evidenced that the inflammatory reaction induced by hyperglycemia activates the PKC pathway which in turn phosphorylates the transient receptor potential vanilloid (TRPV1), present in the plasma membrane of A*δ* and C fibers (nerve fibers for relaying pain). The phosphorylation of TRPV1 results in hypersensitivity of nociceptors and causes pain in diabetic state [35, 36].

By inhibiting PKC in diabetic rats, reduction in hyperexcitability of C-fiber and hyperalgesia was observed. This may be because of the upregulation of the P2X3 receptor in the dorsal root ganglia by PKC in response to the nerve injury. P2X3 is an ionotropic receptor for ATP expressed in the DRG nociceptors [37]. Berberine, a plant alkaloid, was found to relieve PDN in rats by inhibiting TNF-alpha (a proinflammatory cytokine) and modulating PKC*ε* and TRPV1 [36].

### 5.4. AGEs (Advanced Glycation End Products)

Accumulation of advanced glycation end products (AGEs) and their receptor (receptor for advanced glycation end products RAGE) occurs when glucose and other saccharides undergo nonenzymatic reaction which modifies the structure and function of lipids and proteins [25]. Several studies on AGEs have shown that AGEs along with methylglyoxal cause vascular damage. Elevated levels of AGE-RAGE and reduced levels of glyoxalase-1 (which is responsible for the detoxification of methylglyoxal) were reported in type 1 diabetic patients [38]. AGE-RAGE presence has been confirmed in endothelial and Schwann cells. High levels of AGEs cause diabetic neuropathy by elevation of p65 subunit of NF-kB which triggers inflammation and injury in myelinated neuron. AGEs are also associated with induction of apoptosis of Schwann cells [39]. Oxidative stress because of NADPH oxidase activity happens due to neuronal AGE-RAGE interaction [40, 41].

Many recent studies have targeted AGEs in an attempt to ameliorate diabetic neuropathy and painful diabetic neuropathy. Xu et al. reported that the coadministration of AGEs and 1,25-(OH)_2_D_3_ to Schwann cells resulted in suppression of apoptosis (induced by AGEs) through PKA-NF-*κ*B pathway, and it was concluded that vitamin D may be investigated further for diabetic neuropathy [39]. This study complies with the findings by Basit et al. who demonstrated that the intramuscular injection of vitamin D relieves PDN symptoms in the Pakistani population [42]. Therefore, the administration of vitamin D may potentially promote the neuroprotective effects of Schwann cells by neutralizing the catastrophic effects of AGEs. Similarly, interleukin 10 (IL-10, an anti-inflammatory cytokine) was also observed to protect Schwann cells from the effects of AGE via NF-*κ*B pathway [43]. Another research disseminated the partial alleviation of PDN followed by the administration of pyridoxamine in diabetic rats. Pyridoxamine exhibited this activity by inhibiting the effects of RAGE- NF-*κ*B/ERK signaling pathway [44]. Recently, the neuroprotective activity of a medicinal herbal formulation Compound XiongShao Capsule was also published. It was observed that the formulation suppressed thermal and mechanical hyperalgesia significantly by decreasing serum advanced glycation end products, superoxide dismutase, and nitric oxide synthase levels and by inducing apoptosis [45].

### 5.5. Oxidative Stress

The oxidative stress is created intracellularly when there are more free radicals produced than they are being eliminated or utilized by the pathways or enzymes. Oxidative stress plays a key role in diabetic neuropathy. It is mainly caused by free oxygen and nitrogen reactive species like hydroxyl group, hydrogen peroxide, and superoxide. Nitrosative stress is caused due to reactive agents like peroxynitrite and nitrotyrosine which can cause diabetes-induced pain [46]. These free-radicals are generated as a result of shunting of excess glucose to polyol pathway, hexosamine pathway, PKC pathway, and AGE-RAGE interaction [47] which further result in the buildup of cytotoxic metabolites and NADPH overconsumption. These factors combine to enhance intracellular redox stress and aberrant protein, lipid, and DNA changes, resulting in mitochondrial damage and ROS overproduction. The loss of Schwann cells, myelinated axons, and sensory neurons in the dorsal root ganglia brings harm to the peripheral nervous system. Furthermore, inadequate mitochondrial energy generation impairs the ability to transport information down the axons and aggravates axonal damage in diabetic neuropathy. Collectively, oxidative stress in hyperglycemic patient is caused due to increase in lipid peroxidation, GSSH/GSH ratio, 4-hydroxynonenal protein adduct, taurine, and quinine reductase activity in diabetic patients and results in hypoxia, nerve conduction velocity, apoptosis of Schwann cells and neurons, loss of neurotrophic system, and mitochondrial dysfunction. Nrf2 is a transcription factor triggered by the redox status in the milieu and functions to regulate the antioxidant system while NF-*κ*B—another transcription factor—is implicated in the production of inflammatory response. In healthy cells, the regulation of both these factors is coordinated to maintain the redox balance, but in diabetic neuropathy, this balance is disturbed (Ganesh [48]).

A number of agents targeting the oxidative-nitrosative stress have been assessed in the past few decades in an attempt to ameliorate the diabetic neuropathic pain. Berberine has been evidenced to partially bring down the blood glucose level (BGL) and body weight and suppress thermal and mechanical hyperalgesia in diabetic rats. It was suggested that the subject compound ameliorates diabetes and PDN by controlling the elevated oxidative stress and inflammation in the neurons [49]. Likewise, tocotrienol in combination with insulin was observed to reverse PDN in diabetic rats by controlling oxidative-nitrosative stress, caspase 3, and proinflammatory cytokines [46]. Nerunjil (*Tribulus terrestris*) was demonstrated to ameliorate pain threshold in PDN by regulating the oxidative stress and inflammatory response [50]. On the other hand, fisetin was reported to relieve thermal and mechanical pain in diabetic neuropathic rats by normalizing the regulation of Nrf2 and NF-*κ*B [51]. Additionally, *Rosmarinus officinalis L.* has an antinociceptive and neuroprotective effect in diabetic rats, due to its ability to decrease caspase and Bax-Bcl-2 ratio which are the key signaling molecules in causing apoptosis. The neuroprotective activity of the plant is also because of its antioxidant and radical-scavenging activity. Signification effect was seen in the form of reduced thermal hyperalgesia in STZ-induced diabetic rats [52]. Partial reduction in pain has been reported in an animal model of hyperglycemia when treated with kaempferol extracted from *Eruca sativa* [53].

Alpha lipoic acid, a widely tested drug for diabetic neuropathy, appears to slow down or cure it by exhibiting multiple antioxidant activities. Administration of alpha lipoic acid results in an increase in reduced glutathione, which is a vital endogenous antioxidant. A dose of 600 mg alpha lipoic acid was observed to ameliorate neuropathic defects hyperalgesia, numbness, and paresthesia in clinical trials [54–56].

Acetyl L-carnitine has been evidenced to relieve PDN symptoms [57, 58] by a number of mechanisms including antioxidant [59], cytoprotective, and antiapoptotic activity. Its analgesic activity is mediated by bringing down glutamate level in the synapse, contributing majorly via the epigenetic mechanism in which a transcription factor, p65/Rela, of the NF-*κ*B family is acetylated. The acetylated transcription factor then facilitates the upregulation of type-2 metabotropic glutamate (mGlu2) receptors in the DRG and dorsal horn, resulting in a decline in glutamate release from the nociceptors (Di [60, 61]).

### 5.6. PARPs (Poly ADP-Ribose Polymerase)

Under normal circumstances, poly ADP-ribose polymerase (PARP) is involved in repairing DNA and inducing apoptosis. Excessive PARP leads to tissue damage in diabetes mellitus. Hyperglycemic conditions lead to the formation of reactive nitrogen and oxygen species in which single DNA breaks are severed (single-strand DNA break). The consequent upregulation of PARP causes depletion of NAD^+^ in the cell (thus decelerating glucose metabolism and energy generation) and ribosylation of ADP for the production of glyceraldehyde-3-phosphate dehydrogenase (GADPH) because of which vessels supplying the blood to nerves get damage [62]. PARP activation is also associated with nerve conduction deficit in sensory and motor nerves, dysfunction of neurovascular system, gene expression, alteration of transcriptional regulation, and energy failure processes in diabetic animals [63].

In the rodent model of PDN, 1,5-isoquinolinediol (which is a PARP inhibitor) was demonstrated to ameliorate the thermal hyperalgesia, tactile allodynia, and mechanical hyperalgesia [64]. Additionally, 10-(4-methylpiperazin-1-ylmethyl)-2H-7-oxa-1,2-diaza-benzo[de]anthracen-3-one which is another PARP inhibitor when administered orally in the rodent model of PDN resulted in partial alleviation of PDN symptoms along with reduction of intraepidermal nerve fiber degeneration [65].

### 5.7. MAPKs (Mitogen-Activated Protein Kinases)

Mitogen-activated kinases are subdivided into extracellular signal-related kinase (ERK), p38, and c-Jun N-terminal kinase (JNK); all are involved in signal transduction. ERK domains 1 and 2 are associated with neural survival, while JNK and p38 facilitate the neural apoptosis. Upregulation of these three leads to neuropathic pain. JNK upregulation causes phosphorylation of neurofilaments, and downregulation causes neuronal regeneration in diabetic rats [63]. Furthermore, research suggests that the role of long noncoding RNAs in PDN is because of the activation of ERK1/2 and p38 MAPK, and their inhibition may heighten the threshold of thermal and mechanical pain sensitivity, thus relieving PDN [66]. The long noncoding RNAs will be discussed in detail later.

It has been reported that p38 MAPK inhibitors: SB203580 and SD-282, JNK inhibitor: SP600125, and MAPK inhibitor: U0126, have a role in fixing mechanical allodynia and hyperalgesia in diabetic rats [67–69]. It was also reported that berberine exhibits its neuroprotective efficacy in diabetic neuropathy by regulating the MAPK pathway [70] in addition to modulating PKC and inhibiting TNF-alpha in PDN [36].

### 5.8. NF-*κ*B (Nuclear Factor Kappa Light Chain Enhancer of Activated B Cells)

Immune responses and apoptosis are regulated by transcription factors like NF-*κ*B and are triggered by inflammatory stimuli. Studies revealed that the activated NF-*κ*B is found in sciatic, sural nerve and DRG of diabetic animals. NF-*κ*B activity was more prominent in Schwann cells cultivated in higher glucose concentration as compared to cell grown in low glucose medium. Overexpression of p65 subunit of NF-*κ*B causes inflammatory demyelination [71, 72] and oxidative-nitrosative stress which induce insults in the nerve fibers and the vessels supplying blood to these tissues resulting in impaired blood supply and elevated release of inflammatory mediators: prostaglandins and bradykinins. This sequence of events increases the sensitivity to noxious stimulus resulting in neuropathic pain (Ganesh [48]).

Bioactive extracts from Annona reticulata Bark (or) Ziziphus jujuba Root bark have shown positive results in PDN by decreasing the oxidative stress and inhibiting the cascade of NF-*κ*B [73]. Alpha lipoic acid is also found to relieve PDN in diabetic rats by modulating NF-*κ*B cascade and TRPV1 expression [74]. Fisetin has been shown to reduce heat and mechanical stimulus-related hyperalgesia in diabetic animals by restoring Nrf2 and NF-*κ*B regulation [51].

### 5.9. Hh (Hedgehog)

Hh family consists of proteins that usually express in peripheral nervous system and play role in cell growth, cell fate, and survival. The insult to the peripheral nerve initiates a process of degeneration and regeneration during which Hh pathway plays a leading role. The effectors of these pathways Sonic Hedgehog and Desert Hedgehog are involved in the nerve regeneration. Sonic Hedgehog initiates the neovascularization in the vicinity of the injured nerve to facilitate regeneration in diabetic rats [75]. A recent study demonstrated that the upregulation of microRNAs, miR-9 and miR-29a, leads to diabetic neuropathy and the painful symptoms. This was evidenced to be instigated via insulin gene enhancer binding protein-1- (ISL1-) mediated activation of the sonic hedgehog signaling pathway [76]. Decreased Hh levels were seen in diabetic animals causing downregulation of motor neuron conduction velocity, sensory nerve conduction velocity, and reduced pain perception to heat, nerve growth factor and nerve blood flow. But blocking of Hh pathway showed decreased pain perception and neuropathic pain in diabetic rodents, possibly witnessed due to increased endothelial cell permeability, and decreased claudian5 expression [77].

### 5.10. Inflammatory Cytokines

Greater than 30 isoforms of interleukin exist which are classified into anti-inflammatory ILs (IL-4 and IL-10) and proinflammatory (IL-1 beta, IL-6, and IL-8). TNF-alpha is another proinflammatory cytokine that is activated by different immune cells like lymphocytes, natural killer cells, macrophages, and mast cells which confirms that their upregulation produces an immune response. Proinflammatory cytokines are mostly involved in pathogenic signal transduction in diabetic neuropathy. Yu-Wen et al. revealed the substantially elevated levels of IL-6 and TNF alpha in the peripheral nerves and spinal cord of sedentary STZ-induced diabetic rats and the role of these cytokines in PDN [78]. It is well recognized that TNF alpha is also elevated in the human subjects of PDN and the level of TNF alpha is directly related to the severity of pain. The TNF alpha and iNOS immunoreactivity is also prominent and related to pain in PDN patients [79]. The increased level of IL-10 was also noticed in PDN patients, and it is believed that this increment may be the result of the activation of the compensatory mechanism [80, 81].

Minocycline relieves the diabetic neuropathic pain in STZ-treated rats and potentiates the analgesic effects of morphine by upregulating the production of IL-10, IL-2, IL-1 alpha, and sTNF RII. Furthermore, it is believed that minocycline can inhibit PARP and pancreatic beta cell necrosis [82]. It was also observed that neural mobilization (discussed later in detail) in the STZ-induced diabetic rats reduced the mechanical allodynia by cutting the levels of TNF-alpha and IL-1beta [83]. Curcumin derivative J147 is another neuroprotective and powerful neurogenic drug candidate which promotes AMP kinase pathway and inhibits TNF-*α* and other neuroinflammatory markers that cause neurodegeneration, thereby reversing the touch triggered allodynia [84].

### 5.11. COX (Cyclooxygenase)

Two forms of cyclooxygenase enzymes (COX) are reported COX-1, involved in cellular hemostasis and COX-2 which remains silent under normal circumstances and are activated under high glucose level, oxidative stress, PKC activation, and inflammatory cytokines [85]. Studies suggested that COX-deficient rodents are resistant to diabetes related complications like decreased nerve conduction velocity, reduced blood flow around myelin sheath, and diminished nerve fiber density [86].

The combinatorial administration of nimesulide (COX-2 inhibitor) and Rutin (targeting Nrf-2/HO-1) was corroborated to raise the pain thresholds in the diabetic rats [87]. Likewise, celecoxib which is a selective COX-2 antagonist is recognized for countering allodynia and hyperalgesia in diabetic rats. The suggested mechanism of action is the modulation of opioid receptor or voltage-gated sodium and potassium ion channels [88]. The synergistic administration of proglumide (nonselective cholecystokinin (CCK) inhibitor receptor) and celecoxib resulted in a significant reduction of painful symptoms in diabetic rats [88]. Meloxicam, another COX-2 antagonist, is also suggested to relieve allodynia in diabetic rodents [89]. COX-2 inhibitor (SC-58125 and NS-398) when administered intrathecally produced a pronounced antihyperalgesic effect in diabetic animals [90].

Nonsteroidal anti-inflammatory drugs which include ibuprofen, acetaminophen, and aspirin are COX inhibitors and are widely prescribed for relieving pain, but their efficacy is not proven for PDN in humans [91]. The efficacy and safety of the combined administration of Tramadol (an Opioid) and Acetaminophen (an NSAID) were evaluated for PDN. The combination ameliorated the PDN symptoms: pain, sleep quality, mood, anxiety, and quality of life, but the study was discontinued ahead of time because of the adverse outcomes [92]. In another study, it was witnessed that the coadministration of selective serotonin reuptake inhibitors and aspirin increased the risk of gastrointestinal (GI) bleeding [93]. Further, it is suggested that administration of these drugs may also impair the renal function in addition to causing GI bleeding in diabetic patients [94, 95].

### 5.12. NGF (Nerve Growth Factors)

Nerve growth factor as the name suggest is related to nerve propagation and development. Abnormal increase and decrease in NGF concentration can cause serious neuronal damage as it affects many pathways of survival. Some other factors like glia cell-derived neurotrophic factor, brain derived neurotrophic factor, and neurotrophic (NT-3, NT-4, and NT-5), and insulin growth factor I and II are also involved in propagation, angiogenesis, sensitization, and cell growth. NGF is responsible for the propagation of small nerve fiber and sympathetic neurons. Retrograde axonal transportation and NGF-dependent sensory neurons with diminished expression of neuropeptides substance P and calcitonin gene-related peptide (SP, CGRP) are affected by the absence of TRKA (tropomyosin receptor kinase A) in hyperglycemia (Tomlinson, Fernyhough, and Diemel 1997). In diabetic rats, reduced skin fiber through SP was found in association with decreased level of NGF which can act as an evidence for developing polyneuropathy other than vascular and metabolic changes. NGF also affects the developmental and regulatory pathways of cardiac nervous system.

In addition, in PDN, the cutaneous neurotrophin nerve growth factor (NGF) level rise. NGF gives rise to mechanical allodynia in mouse models [96], and NGF/p38 signaling enhances intraepidermal nerve fiber density (IENFD) in PDN [96, 97]. NGF stimulates cutaneous nociceptors in humans and is thought to be the source of hypersensitivity and hyperalgesia in PDN [98]. It has a two-edged effect. NGF levels in the dorsal root ganglion (DRG) and dorsal horn in rat models, when explored, were discovered to drop in the DRG 1 week after diabetes induction and in the dorsal horn 2 weeks after diabetes induction. Hyperalgesia is caused by decreased NGF expression in the DRG, while allodynia is caused by decreased NGF expression in the dorsal horn of the spinal cord. Exogenous NGF has been shown to alleviate diabetic neuropathic pain [99]. But it is already known that the administration of endogenous NGF in PDN patients resulted in pain relief in phase II trial but failed to perform better than placebo in the phase III trial [100–102].

### 5.13. Autophagy

Autophagy is a metabolic pathway triggered by oxidative stress in which cytoplasmic materials are sent to the lysosome for breakdown and reuse of the by-products. It comprises molecules which are key players of recycling pathways and maintain cellular homeostasis. Autophagosomes, a double membrane bounded vesicle, are formed when a phagophore internalizes a damaged component from the cytoplasm. Degradation machinery is a combination of autophagosomes and lysosomes [103]. The downregulation of autophagy in PDN is mediated by P13K/AKT/mTOR. It was found that autophagy downregulation in spinal cord could play a role in the etiology of PDN while the maintenance of PDN is somewhat aided by increased autophagy in the spinal cord. It was revealed that rapamycin injection reduced the mechanical pain threshold in diabetic rats. The expression of LC3-II (biomarker of autophagy) and Beclin1 protein (well known to trigger autophagy) was considerably higher in the spinal cords of rapamycin-treated diabetic rats than in nonsupplemented diabetic rats [104, 105].

In diabetic rats, inhibiting the PI3K/AKT/mTOR pathway increases autophagy and alleviates hyperalgesia [104]. *Lycium barbarum* polysaccharide heightened the nociceptive thresholds in diabetic rats by inhibiting of mTOR/p70S6K, thereby augmenting autophagy [106].

### 5.14. GSK3 (Glycogen Synthase Kinase 3)

Glycogen synthase kinase 3 (GSK3) facilitates the addition of phosphate on serine and threonine amino acid. Diverse genes encode for GSK3-alpha and GSK3-beta. Pathways that it controls involve migration, apoptosis, cellular proliferation, and glucose regulation. Neuronal anterograde axon transportation has been also found to be lined with GSK3. Peripheral and central inflammatory responses are governed by GSK3-beta (King et al. 2015). In diabetic rats, the mRNA of GSK3-beta is upregulated and it was found that the nonpharmacological endurance training of the rats having PDN can regulate this increment [107].

### 5.15. Pyruvate Dehydrogenase Kinases (PDKs)

In normal metabolism, there is a balance between nutrient intake and consumption. There are two distinct routes for metabolism of fatty acids and glucose that converge just before the TCA cycle. Briefly, fatty acid converts to Acyl-CoA in the cytosol which then moves inside the mitochondria for further metabolism. Fatty acid oxidation in the mitochondria results in the conversion into acetyl-CoA. On the other hand, glucose metabolizes to pyruvate in glycolysis which then moves into the mitochondria and experiences oxidative decarboxylation to form acetyl CoA under the action of mitochondrial gatekeeping enzyme pyruvate dehydrogenase complex (PDC). Acetyl CoA in both cases (fatty acid and glucose metabolism) is, subsequently, used in TCA cycle. Pyruvate dehydrogenase kinases (PDKs) may phosphorylate the PDC and inhibit its action. Upon inhibition of PDC, the excess pyruvate is covered to lactic acid [108].

PDK is believed to be abnormally upregulated in dorsal root ganglion (DRG) cells in PDN; this may be due to the hypoxia in the DRG. The cells in the DRG include the neuronal cell bodies, satellite glial cells, and the infiltrating macrophages. The surge in lactic acid as a result of the induction of PDKs contributes to the pathogenesis of PDN by triggering reactive gliosis, macrophage infiltration, acidic microenvironment, proinflammation, and sensitization of the peripheral neurons which ultimately results in central sensitization and pain hypersensitivity [109]. In a study published in 2016, it was witnessed that the genetic ablation of PDK2 and PDK4 mitigated PDN in the streptozotocin-induced diabetic rats and it was concluded that the glucose-PDK2/4-PDC-lactate axis in the DRG may serve as a potential pharmacotherapeutic target for PDN [109].

### 5.16. Satellite Glial Cells (SGCs)

Satellite glial cells are present in the sensory, parasympathetic and sympathetic ganglia, and functions to envelop the neuronal cell bodies. The activation of SGCs in sensory ganglia may contribute to PDN via a number of ways: altered enzymatic activity in SGCs (aldose reductase, PDK2 and PDK4) [109, 110], upregulation of P2X4R and P2X7R [111, 112], and excitation of nociceptors by SGC-derived cytokines [113, 114].

### 5.17. Long Nonprotein Coding RNA

The long nonprotein coding RNA NONRATT021972 is upregulated in pathogenesis of diseases of nervous system, and it was evidenced to be upregulated in PDN as well. In DRG SGCs of diabetic rats, BzATP-activated currents are substantially higher than in control rats. When the effect of small interfering RNA (siRNA) for NONRATT021972 was assessed, it was found that the intravenous injection of NONRATT021972 siRNA resulted in downregulation of P2X7, TNF-alpha, and glial fibrillary acidic protein (GFAP). Further, the ATP-activated currents and resultant diabetic neuropathy pain symptoms were reduced by NONRATT021972 siRNA treatment [111]. Similarly, uc.48+ siRNA and BC168687 siRNA alleviate the PDN symptoms by downregulating the levels of proinflammatory cytokines [66].

## 6. Topical Agents

Topical analgesic agents for painful diabetic neuropathy include lidocaine, capsaicin, and nitric oxide sprays [17]. The effects of these drugs are discussed in detail in this section.

### 6.1. Lidocaine

Lidocaine is a topical agent imparting the antagonistic effect on the sodium-gated voltage channels, i.e., Na_v_ 1.7 and Na_v_ 1.8. It is recommended by the American Association of Clinical Endocrinologists (AACE) and American Academy of Neurology (AAN) for the management of pain in PDN patients [115]. It helps in stabilizing the membrane potential of the small nerve fibers by retaining hyperexcitability causing reduced release of neuronal active substances. Studies regarding lidocaine are being conducted; for example, the application of lidocaine in healthy subjects resulted in a substantial change in the thresholds of the pain stimulus relayed by small fibers. Small nerve fibers are associated with the pain as a result of thermal and mechanical stimulus. It was observed that this effect was due to the partial blockage of these nerve fibers [116]. In another study, the safety and efficacy of the 5% lidocaine patches were assessed and it was confirmed that the application of about four patches in a duration of 18 hours per day was effective in alleviating pain and improving life quality and was well tolerated in PDN patients [117]. Pharmacodynamics and pharmacokinetic studies conducted on rat models with diabetic neuropathy showed that as compared to local anesthesia, lidocaine patch showed lower inhibitory concentration for blocking of sodium ion channels in the nerve fibers associated with pain conduction to CNS (Ten [118]). Meta-analysis and systematic review studies have shown that 5% lidocaine patch has the similar pain reduction capacity when compared with pregabalin but has a better safety profile [119].

### 6.2. Nitrates

Topical nitrates are not recommended in any of the guidelines for treating PDN, but they are used off-label [115]. A randomized, placebo-controlled, double-blind study showed promising results in reducing overall neuropathic symptoms (*p* = 0.02) along with the burning sensation (0.006) with the usage of isosorbide dinitrate spray [120, 121]. Study involving the administration of L-arginine to the rat model of PDN showed a decrease in thermal and tactile allodynia and mechanical hyperalgesia by regulating plasma level of nitric oxide [122]. A study by Quattrini et al. highlighted a reduced sympathetically mediated vasoconstriction in the foot of PDN patients. It was anticipated that the local sympathetic dysfunction may induce a heightened cutaneous shunting and compromised dermal nutritional blood flow, resulting in hypoxia which may trigger PDN symptoms ultimately. Correction of this condition could be the possible mechanism of action by which pain relief is imparted by local vasodilators, isosorbide dinitrate patches, and glyceryl trinitrate spray [123].

## 7. FDA-Approved Drugs

The FDA-approved drugs for painful diabetic neuropathy include pregabalin, duloxetine, tapentadol, and capsaicin (for foot pain only). These are discussed below in detail.

### 7.1. Duloxetine

Duloxetine inhibits the reuptake of neurotransmitters, i.e., norepinephrine and serotonin, and shows less affinity towards dopamine transporters. Having lesser to no affinity for glutamate, dopaminergic, opioid, adrenergic, GABA, cholinergic receptors, and no inhibitory action on monoamine oxidase, duloxetine is simply classified as SNRI (selective norepinephrine-serotonin reuptake inhibitor) [124]. Therefore, the mechanism through which duloxetine lowers diabetic neuropathy pain is through inhibition of reuptake of serotonin-norepinephrine. As SNRIs affect the neurotransmitter balance in the brain, this indicates that their central pain inhibitory action may be due to the inhibition of noradrenergic and serotonergic neuronal activity. Reduction in these inhibitory signaling may lead to persistent pain perception in the brain. Both neurotransmitters (serotonin and norepinephrine) play a pivotal role in relaying pain signals in spinal cord and brainstem. Synergistic inhibition of these neurotransmitters causes less transmission of pain signals from periphery to CNS. A number of preclinical and clinical trials have shown increased tolerance to pain with reduced pain symptoms in diabetic-induced painful neuropathy [125–127]. Pathogenic mechanism involving NF-*κ*B can cause spinal glial cells to secrete active substances and cytokines that can cause a sustained neuropathic pain. Duloxetine (SNRI) reportedly inhibits secretion of NF-*κ*B and TLR4 in dorsal root ganglion of rat [128]. Inactivation of microglial secretion has shown a neuroprotective and restoring effect on peripheral nerve injury with increased levels of nerve growth factors especially in sciatic nerve of diabetic rats [126]. Comparative double blinded study was conducted with gabapentin and duloxetine which suggested that the former shows more side effects whereas the later shows more medication compliance [129].

### 7.2. Gama Aminobutyric Acid (Pregabalin)

Health experts prescribe gabapentinoid drugs: pregabalin and gabapentin for the management of peripheral and central neuropathies. Gabapentinoids are considered the first line of treatment in mitigating these complications. These are analogues of GABA and are known to bind with auxiliary subunits of calcium channels (*α*_2_*δ*-1 and *α*_2_*δ*-2). Pregabalin does not exhibit any interaction with GABA A and B receptors or triggers GABA uptake [130]. The potency ratio of pregabalin is higher than gabapentin, as the former has more affinity towards *α*_2_*δ*-1 subunit [131]. For the management of painful diabetic neuropathy, pregabalin is a first line of treatment according to international guidelines. Dose-dependent studies have been conducted on pregabalin drug to quantify the pain intensity in diabetes-induced neuropathies [125, 132, 133]. Pain reduction was less than 50% in 3 : 10 patients taking 300/600 mg pregabalin daily as compared to 2 : 10 with placebo. With 600 mg daily dose, patients reported somnolence 15% and dizziness 22% [134]. In another research, a comparative analysis between venlafaxine, carbamazepine, and pregabalin was done, showing pregabalin more potent in curing painful diabetic neuropathy [135]. Researchers have documented that 600 mg/d pregabalin is well tolerated, reduces pain significantly, and does not affect the nerve conduction velocity [136].

On the other hand, gabapentin has been reported in treating neuropathies in animal models, but it is not approved by the FDA for the treatment of diabetic neuropathic pain. It is widely used and recommended in other guidelines for PDN [137]. It synergistically interacts between elevated GABA production, non-NMDA receptor inhibition, and strong affinity for alpha-2-delta subunit of voltage-gated calcium channel [138]. Gabapentinoids interacts with the highest-affinity binding sites present in the brain membrane. In the *in vitro* studies, it has been found that these gabapentinoids modulate the activity of GABA synthase enzyme, glutamic acid decarboxylase (GAD), and glutamate synthase, branched amino acid chain transaminase [139]. A study was conducted by Andrew Moore *et al*. to check the effect of gabapentin on 5914 patients. According to the study, gabapentin (1200 mg/day) has been proven efficacious in treating painful neuropathies—38% patients reported reduction in pain by 50% vs placebo [140]. A novel study involving coadministration of gabapentin and tramadol showed a synergic effect on neuropathic pain reduction by IL-1*β* proinflammatory suppression in the mouse model. The effect was not tested on the PDN model, but this study indicated that gabapentin can be combined with other drugs to increase its effectiveness in managing painful symptom [141]. Carbamazepine, another anticonvulsant, has exhibited effectiveness against different neuropathic syndromes and especially PDN [142]. But its efficacy is comparatively lower than that of pregabalin [135], and it is not recommended for PDN in international guidelines.

The effectiveness of pregabalin against diabetic neuropathic pain may be because of its antidepressant nature. PDN is a major determining factor of depression and is significantly associated with it [143–145]; noradrenergic antidepressants and gabapentinoid anxiolytics are antineuropathic medications that have been licensed and/or recommended for PDN [146]. Pregabalin is approved to treat neuropathic pain and PDN, and it may also be used to treat concomitant anxiety and sleep disturbances [147]. It may have an antinociceptive effect on its own, but this is still subject to debate [148].

### 7.3. Opioids (Tapentadol Extended Release)

Tapentadol is the first representative of a class of drugs referred to as mu-opioid receptor agonist/noradrenaline reuptake inhibitor (MOR-NRI) drugs, i.e., centrally acting analgesic drugs. It is FDA approved, is taken orally, and has analgesic and noradrenergic properties making it effective towards managing pain symptoms [149]. Clinical trials have shown positive result of tapentadol in managing diabetic peripheral neuropathy and chronic lower back pain. Tapentadol has dual mechanism of action, i.e., u-opioid receptor agonism and norepinephrine reuptake inhibition contributing to its antineuropathic potential in the substantia gelatinosa of spinal cord [150]. Tapentadol extended release has found to have some adverse effects: nausea, vomiting, headache, somnolence dizziness, and constipation. Long-term opioid administration greatly increases the risk of addiction [151]. Based on their poor tolerance by the body and safety concerns associated with their use, opioids are considered the second line of treatment in neuropathic pain management [152].

Lastly, one of the characteristic properties of tapentadol is its minimal serotoninergic activity which is beneficial for pain management in patients. Studies related to tapentadol-extended release are being done to check its neuroprotective effect. In a study, patients were titrated with dose of tapentadol ER 100-250 mg twice a day, following which the changes in pain intensity were reported in placebo 1.28 (2.41) and tapentadol ER 0.08 (1.87) on 11-point numerical rating scale. Adverse effects associated with placebo were 56.0% and with tapentadol ER 74.7%. Results obtained from this pooled analysis exhibited analgesic efficacy of tapentadol ER in managing painful diabetic neuropathy. An important point to consider is that the results were consistent among different PDN subcategories in terms of treatment effectiveness [153]. Large-scale, phase 3 studies showed that tapentadol ER was well tolerated among the patients suffering from chronic osteoarthritis, lower back pain, and painful diabetic peripheral neuropathy [154].

### 7.4. Capsaicin

Topical capsaicin is approved by the FDA only for relieving the foot pain in PDN [155]. It is a TRPV1 agonist which triggers depolarization in the nociceptor by facilitating the influx of Na^+^ and Ca^++^ and by triggering the release substance P. Recurrent exposure of TRPV1 to capsaicin results in the scarcity of substance P and renders desensitization and inactivity of TRPV1 [17]. The 8% capsaicin patch has been tailored to deliver high levels of capsaicin rapidly, which facilitates the inhibition of the hyperexcited nociceptor, thus tampering the ectopic release of nerve impulse [156]. It has been proven that the application of 8% patch for a period of 30 minutes relieves pain and ameliorated the life quality in PDN population [156]. However, the lower concentration of capsaicin gel 0.025% showed insignificant results in managing painful symptoms in patients with PDN but was safe and well tolerated as compared to higher concentration of capsaicin [157]

## 8. Nonpharmacological Treatment Modalities for PDN

### 8.1. Exercise

It was observed that physical activity in diabetic rats alleviated the PDN symptoms at least temporarily by increasing the levels of heat shock protein 72 (Hsp72) [78]. Neurodynamics or neural mobilization is a treatment modality that mobilizes the nervous system and/or the structures encompassing it through exercise or manual methods. The aim in this intervention is to reinstate the homeostasis of the nervous system and its associated structures. A number of preclinical and clinical studies have demonstrated the effectiveness of this intervention in restoring the fluid dispersion within the neuron and immune reaction and in healing the intraneural edema and thermal and mechanical hyperalgesia [158]. It STZ-induced diabetic rats, it was revealed that the neural mobilization relieved the mechanical allodynia by cutting the levels of TNF-alpha and IL-1beta [83].

### 8.2. Spinal Cord Stimulation (SCS)

SCS is an invasive modality for treating chronic algesia that involves activation of the dorsal columns of the spinal cord using a low-voltage electric current. The mechanism of action remains elusive; however, this intervention is thought to affect both spinal and supraspinal regions. In most cases, the SCS device is implanted in two stages. The electrode lead is first inserted percutaneously in the epidural space and attached to a temporary pulse generator external to the body (the trial phase). The external pulse generator is only replaced by an implanted pulse generator if the treatment results in significant pain reduction; otherwise, the lead is removed and no SCS therapy is offered [159, 160].

SCS was shown to improve the pain symptoms and life quality of 60 PDN patients for a period of 6 months [160]. Another recent study revealed the success of this intervention for 86% of PDN patients after 1 year of initiation of SCS treatment and in 55% of PDN patients after 5 years [161]. Recently, FDA accorded premarket approval for senza spinal cord stimulation system for the treatment of diabetic neuropathy pain.

## 9. Discussion

Diabetic neuropathy is a complex and grave nerve degenerative disorder, which affects 40-80% of people suffering from diabetes globally. Signaling pathways are essentially responsible for the initiation and pathogenesis of this disorder. Whereas comprehensive studies are required on transcriptional and translational levels to understand the exact development and regulation of these pathological mechanism causing difficulty in developing exact treatment for diabetic neuropathy, slowing down this pathological mechanism is the main aim for the symptomatic based management of painful diabetic neuropathy. For the attenuation of painful symptoms, several tricyclic antidepressants, anticonvulsant, and opioids are prescribed to diabetic patients. Curative effect of medicine is dependent on the way they cause the suppression/activation of neuropathic signaling/inhibiting molecules. Evidence from clinical and preclinical studies shows that neuropathic suppression of signaling molecules via aldose reductase, MAPKs, PKC isoforms, and oxidative stress has proven effective in dealing with painful symptoms of diabetes. These have been proven beneficial in enervating nerve damage. Other than the above-mentioned pathway initiation of neuropathic pathology inhibition through PARPs, siRNAs, and inhibitors of hedgehog pathway need to be investigated further. Currently, the management of painful diabetic neuropathy is achieved locally as well as systemically. The commercially available drugs for relieving diabetic neuropathic pain involving localized treatment are capsaicin, 5% lidocaine patch, and nitrates spray whereas drugs for systemic suppression of diabetic neuropathic pain are gabapentinoids (pregabalin and gabapentin) duloxetine and opioids. The recent FDA approval of the spinal cord stimulation system also seems promising in relieving this agonizing disorder called PDN. Furthermore, large-scale clinical trials with comparative analysis of existing and novel pharmacotherapeutic agents are required in order to develop a more localized and potent therapeutic alternative with fewer side effects. In this regard, building understanding regarding the mechanisms involved in painful diabetic neuropathy, molecular targets, and devising a novel drug (inhibitors or suppressors)/drug delivery system should be the researcher's approach in managing diabetes induced painful neuropathy in the future.  Table 1 summarizes the mechanism of action of the pharmacotherapeutic agents that have been investigated preclinically or clinically so far. It also includes the FDA-approved drugs for PDN, and the topical agents available in the market that are used for PDN.

## 10. Conclusion

Painful diabetic neuropathy has high prevalence, is under diagnosed, requires expensive treatments, and lacks effective therapy. Lack of understanding regarding the pathogenesis of painful diabetic neuropathy is the main reason behind the shortfall of its treatment whereas management of this disease is being done by symptomatic pain management, glycemic control, risk factor management, and pathogenic mechanism-based management. Currently available therapies which significantly reduce diabetes-induced painful neuropathies mainly include agents that work on ion channels; i.e., pregabalin antagonizes calcium channels. Recent ongoing research is targeting mechanisms, e.g., polyol pathway, hexamine pathway, PKC, oxidative stress, PARP, MAPK, AGE, NF-KB, Hh, COX, IL, TNF-alpha, NGF, autophagy, and GSK3 which either inhibit or activate molecules responsible for either signaling or suppressing these pathological mechanisms. Therefore, mechanism-based approaches should be the way forward in tackling diabetes induced painful neuropathies. However, current agents (duloxetine, opioids, r-aminobutyric acid, etc.) have limited efficacy and possess intolerable side effects in the long run. Adjuvant therapies are also efficacious but are under moderate clinical use. Furthermore, clinical trials on large scale with comparative analysis among already existing drugs and novel drugs need to be done to develop a localized and more potent treatment option with minimal side effects.

## Figures and Tables

**Table 1:** List of therapeutic drugs involved in management of PDN. (-) shows decrease in the level of that component, and (+) shows increase in the level of that component.

Sr	Drugs	Target	Observation	Clinical or Preclinical	References
1	Epalrestat	Polyol pathway	Spontaneous pain (-), MNCV (+), SNCV (+), vibration perception threshold (+), F-wave latency (-)	Clinical and preclinical	[22, 162]
2	Sorbinil	Polyol pathway	Polyol pathway (-), Na pump defect (-), defective axonal transport (-), NCV (+), myelinated fiber repair (+)	Preclinical and clinical	[163, 164]
3	Fidarestat	Polyol pathway	Sorbitol accumulation (-), spontaneous pain (-), median nerve FCV (-), minimal latency (-), NCV (+)	Clinical	[14, 165]
4	Zenarestat	Polyol pathway	Nerve conduction velocity (NCV) (+), sorbitol in sciatic nerve (-)	Clinical and preclinical	[165, 166]
5	Tolrestat	Polyol pathway	MNCV (+), polyol influx in nerve (+), neuropathic pain (-)	Clinical	[167]
6	Benfotiamine	Hexosamine pathway	Pain (-) when coadministered with alpha lipoic acid	Clinical	[32]
7	Berberine	PKC pathway, MAPK pathway, TNF-alpha, oxidative stress, TRPV1	Thermal hyperalgesia (-), mechanical hyperalgesia (-)	Preclinical	[36]
8	Vitamin D	AGE	Pain (-) neuroprotective effect on Schwann cells (+),	Clinical and preclinical	[39, 42]
9	Pyridoxamine	RAGE/NF-kB/ERK	Mechanical allodynia (-)	Preclinical	[74]
10	Compound XiongShao Capsule	AGEs	Thermal hyperalgesia (-), mechanical hyperalgesia (-),	Preclinical	[45]
11	Tocotrienol	ROS	Reversed PDN when administered in combination with insulin	Preclinical	[46]
12	Tribulus terrestris extract	ROS, inflammatory mediators	Pain threshold (+)	Preclinical	[50]
13	Fisetin	ROS, NF-*κ*B,	Thermal and mechanical pain (-)	Preclinical	[51]
14	*Rosmarinus officinalis L.*	ROS	Antinociceptive (+), anti-neuropathic (+)	Preclinical	[52]
15	Kaempferol extracted from Eruca sativa	ROS	Partial pain reduction	Preclinical	[53]
16	Alpha lipoic acid	NF-*κ*B, ROS, TRPV1	Hyperalgesia (-), reduced glutathione (+)	Clinical	[54–56]
17	Acetyl L-carnitine	ROS	Mechanical allodynia (-), synaptic glutamate level (-), NCV (+), nerve regeneration (+)	Clinical	[58, 60, 61]
18	1,5-Isoquinolinediol	PARP inhibitor	Thermal hyperalgesia (-), tactile allodynia (-), mechanical hyperalgesia (-)	Preclinical	[64]
19	10-(4-Methylpiperazin-1-ylmethyl)-2H-7-oxa-1,2-diaza-benzo[de]anthracen-3-one	PARP inhibitor	Intraepidermal nerve fiber degeneration (-), partial reduction of pain (+)	Preclinical	[65]
20	SB203580	p38a MAPK inhibitors	Mechanical allodynia (-), hyperalgesia (-)	Preclinical	[67–69]
21	SD-282				
22	SP600125	JNK inhibitor			
23	U0126	MAPK inhibitor			
24	Ziziphus jujuba Root bark	NF-*κ*B, ROS	Thermal hyperalgesia (-), mechanical hyperalgesia (-), cold allodynia (-)	Preclinical	[73]
25	Desert Hedgehog deficiency	Hedgehog pathway	Thermal hyperalgesia (-)	Preclinical	[77]
26	Minocycline	Cytokines, PARP	Neuropathic pain (-) in combination with morphine	Preclinical	[82]
27	Curcumin derivative J147	AMP kinase pathway, TNF-*α*	Touch triggered allodynia (-)	Preclinical	[84]
28	Nimesulide	COX-2	Pain threshold (+) when administered in combination with rutin	Preclinical	[87]
29	Celecoxib	COX-2	Allodynia (-), hyperalgesia (-)	Preclinical	[88]
30	Meloxicam	COX-2	Allodynia (-)	Preclinical	[89]
31	SC-58125 and NS-398	COX-2	Hyperalgesia (-)	Preclinical	[90]
32	Endogenous NGF	NGF	Pain relief in phase II, but no statistically significant pain relief in phase III trials	Clinical	[100–102]
33	Exogenous NGF	NGF	Mechanical pain threshold (+)	Preclinical	[99]
34	Lycium barbarum polysaccharide	Autophagy, mTOR/p70S6K,	Pain thresholds (+)	Preclinical	[106]
35	NONRATT021972 siRNA	Long nonprotein coding	ATP activated currents (-), spontaneous pain (-), P2X7 (-), TNF-alpha (-), GFAP (-)	Preclinical	[111]
36	uc.48+ siRNA	Long nonprotein coding	Spontaneous pain (-), proinflammatory cytokines (-)	Preclinical	[66, 168]
37	BC168687 siRNA	Long nonprotein coding		Preclinical	[169]
*FDA/EU-approved drugs*					
1	Pregabalin	*α*_2_-*δ* ligand	Neuropathic pain (-)	Clinical	[136]
2	Duloxetine	SNRI inhibitor	Neuropathic pain (-)	Clinical	[124]
3	Tapentadol ER	Mu-opioid receptor agonist and norepinephrine reuptake inhibitor.	Pain reduction (+)	Clinical	[153]
4	Capsaicin	TRPV1 agonist	Pain sensitivity (-)	Clinical	[156]
*Topical drugs*					
1	Lidocaine	Blockers of voltage-gated Na^+^ channels	Na^+^ ion influx (-), pain transduction pathway (-)	Clinical	[117]
2	Nitrate	NO donor	NO (+), vasodilation (+)	Clinical	[121]

MNCV: motor nerve conduction velocity; NCV: nerve conduction velocity.

## Abbreviations

12(S)HETE: 12-Hydroxyeicosatetraenoic Acid
15(S)HETE: 15-Hydroxyeicosatetraenoic Acid
ACE: Angiotensin-converting enzyme
AGE: Advanced glycation end products
AGE-RAGE: Advanced glycation end product-receptor for advanced glycation end products
AMP: Adenosine monophosphate
AR: Aldose reductase
ATP: Adenosine triphosphate
Ca^+2^: Calcium ion
CGRP: Calcitonin gene-related peptide
COX: Cyclooxygenase
DAG: Diacylglycerol kinase
DNA: Deoxyribose-adenine-dinucleotide
DRG: Dorsal root ganglion
ERK: Extracellular signal-regulated kinases
FDA: Food Drug Authority
GABA: Gamma-aminobutyric acid
GAD: Glutamic acid decarboxylase
GADPH: Glyceraldehyde 3-phosphate dehydrogenase
GSK3: Glycogen synthase kinase 3
GSSH/GSH: Glutathione oxidized state/glutathione-reduced state
Hh: Hedgehog pathway
HMG- CoA: 3-Hydroxy-3-methylglutaryl-CoA
IL: Interleukin
JNK: c-Jun N terminal kinase
LOX: Lipoxygenase
MAPK: Mitogen-activated protein kinases
MOR-NRI: Mu-opioid receptor agonist/noradrenaline reuptake inhibition
Na^+^: Sodium ion
NAD^+^: Nicotinamide adenine dinucleotide
NADPH: Nicotinamide adenine dinucleotide phosphate
NF-*κ*B-Kappa: Light-chain-enhancer of activated B cell
NF-*κ*B-P65-Kappa: Light-chain-enhancer of activated B cell-transcription factor p65
NGF: Nerve growth factors
NO: Nitric oxide
NT: Neurotransmitter
PAI-1: Plasminogen activator inhibitor type 1
PARP: Poly-ADP ribose polymerase
PKC: Protein kinase C
SP: Subunit protein
STZ: Streptozotocin
SIRT2: NAD^+^-dependent protein deacetylase sirtin-2
TNF-*α*: Tumor necrosis factor alpha
TRKA: Tropomyosin receptor kinase A
VEGF: Vascular endothelial growth factor.
